# Adhesive force measurement of steady-state water nano-meniscus: Effective surface tension at nanoscale

**DOI:** 10.1038/s41598-018-26893-5

**Published:** 2018-05-31

**Authors:** Soyoung Kwon, Bongsu Kim, Sangmin An, Wanhee Lee, Ho-Young Kwak, Wonho Jhe

**Affiliations:** 1 Department of Physics and Astronomy, Institute of Applied Physics, Seoul National University, Seoul, 08826 Korea; 2 Mechanical Engineering Department, Chung-Ang University, Seoul, 06974 Korea

## Abstract

When the surface of water is curved at nanoscale as a bubble, droplet and meniscus, its surface tension is expected to be smaller than that of planar interface, which still awaits experimental studies. Here, we report static and dynamic force spectroscopy that measures the capillary force of a single nanoscale water meniscus at constant curvature condition. Based on the Young-Laplace equation, the results are used to obtain the effective surface tension (ST) of the meniscus, which decreases to less than 20% of the bulk value at the radius-of-curvature (ROC) below 25 nm, while indicating the bulk behaviour above ~130 nm ROC. Interestingly, such a possibility provides a qualitative resolution of the unsettled discrepancies between experiments and theories in the thermodynamic activation processes for the mentioned three types of nano-curvatured water. Our results may not only lead to development of microscopic theories of ST as well as further experimental investigations, but also help better understanding of the ST-induced nanoscale dynamics such as cluster growth or protein folding, and the ST-controlled design of nano-biomaterials using the nano-meniscus.

## Introduction

Surface tension (ST) is one of ubiquitous physical quantities that play a critical and central role in wide areas of science and engineering, ranging from nucleation of nano-bubbles to stabilization of proteins to controlled surface accumulation of compounds. Therefore, the microscopic understanding of ST is of much interest and importance because it consists of the long-standing and controversial questions concerning the physical characteristics of ST at nanoscale in contrast to bulk^1–3^. The first thermodynamic treatment of the spherical gas-liquid interface was given by Gibbs, which was extended by Tolman who obtained a widely-used, qualitative formula for the slight decrease of ST at the molecular-size scale^4–6^.

**Figure 1:**
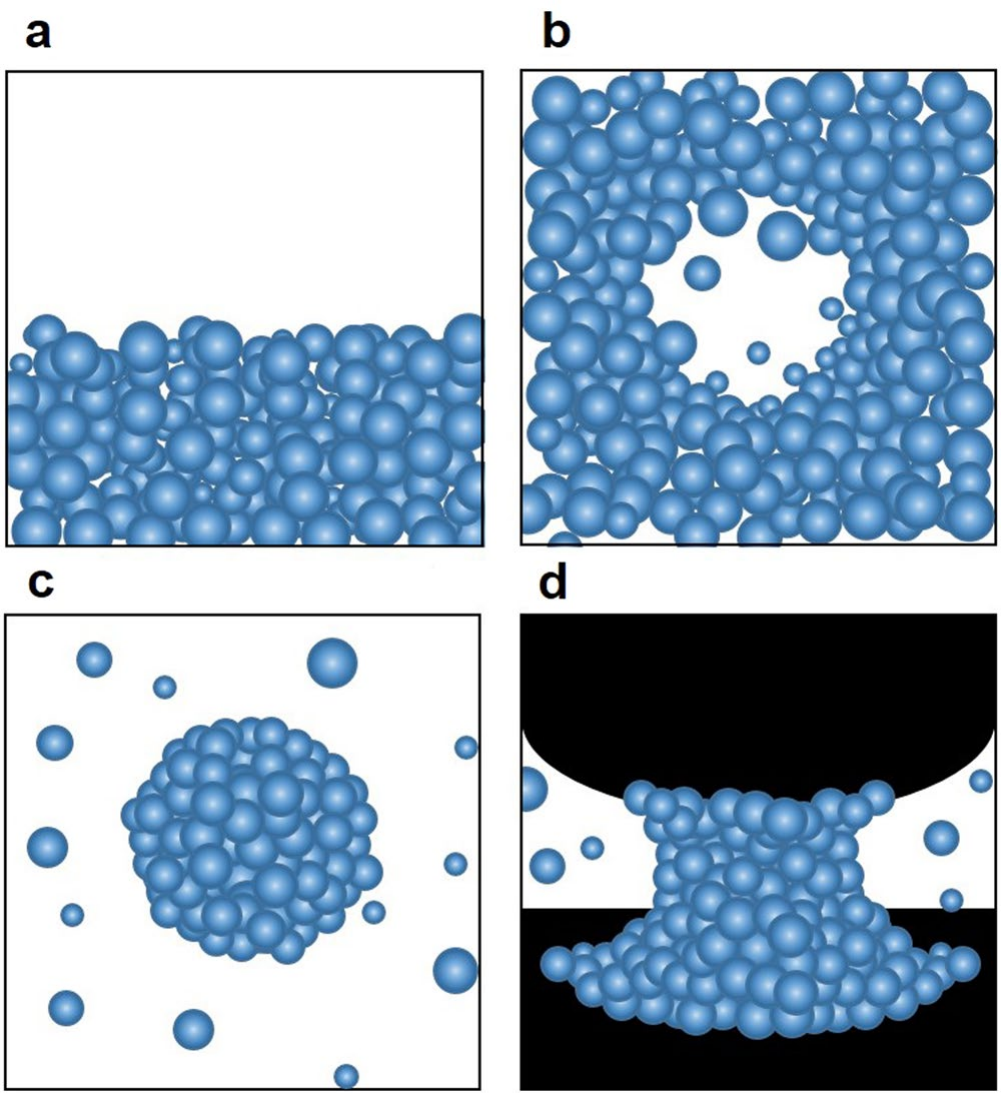
Various forms of nanoscale water and surface tension. (**a**), On the planar liquid-vapour interface, ST assumes the bulk value, *γ*_bulk_. (**b)**, On the nano-bubble, (**c)**, on the nano-droplet, and (**d)**, on the nano-meniscus, the strength of the ST force is, in general, smaller than that of the planar case, and consequently ST at nanoscale is expected to be decreased below *γ*_bulk_, which is still an experimental challenge.

Despite extensive theoretical studies on the curvature-dependent ST reported thenafter^3–8^, however, it has been difficult to quantify such a dependence beyond the classical Tolman results, and there are still on-going debates on the validity of bulk ST (Fig. 1a) for nanoscale water. Moreover, there have been noticeable discrepancies between experimental results and theoretical predictions for the liquid-vapour phase-transition processes observed in the typical nanoscale systems of water (Fig. 1b,c,d), which remains unresolved and yet to be established experimentally^3–9^. Therefore, a well controlled experiment that quantifies the ST at nanoscale is in high demand, where the full-range and accurate capillary force measurement can be made. For this purpose, the water nano-meniscus is desirable due to its uniquely proven stability, controllability and force-sensitivity. Using the validity of the Young-Laplace equation at nanoscale^10^, our results of the measured total forces can be understood in terms of change of the ‘effective’ ST of water (refer to further discussions later).

Here, we employ the hybrid force-measurement system^11^ that combines a tapping-mode, amplitude-modulation atomic force microscope (AFM) and a microelectromechanical system (MEMS). It detects simultaneously the full-range dynamic as well as static forces associated with the capillary-condensed water nano-meniscus^11^. The values of the effective ST for the nanoscale menisci are derived by direct comparison of the measured interaction forces and the capillary force fitting based on the Young-Laplace equation^12,13^, whose validity at nanoscale has been justified by the accurate surface-force experiment^14^. We (i) observe gradual increase of the effective ST with ROC up to 25-nm ROC, and (ii) show how the effective ST-based analysis provides a possible resolution of the afore-mentioned discrepancies (see discussion below)^14,15^.

**Figure 2:**
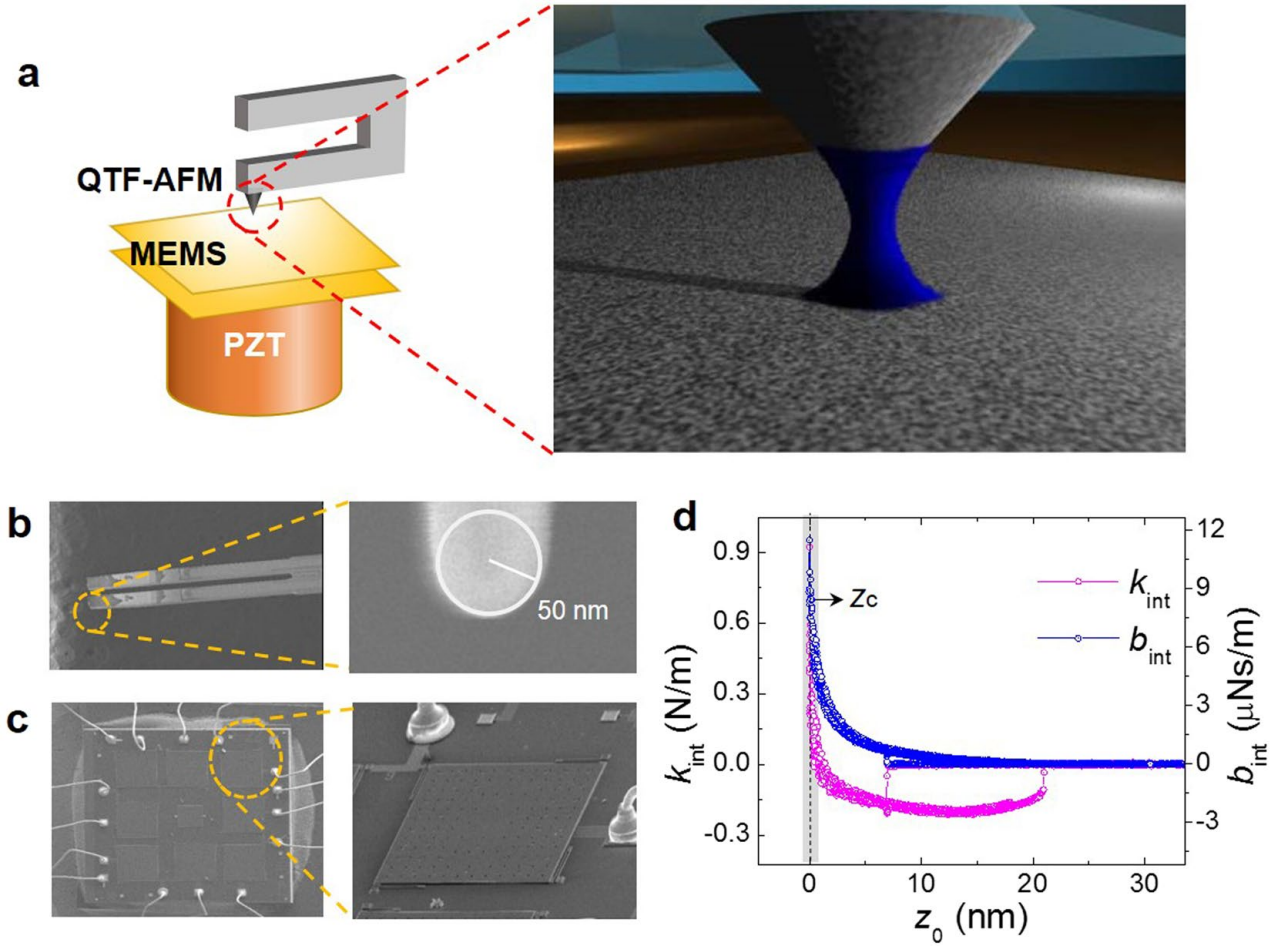
AFM-MEMS system for full-range capillary force measurement. (**a**), Experimental scheme of the force-measurement for the nanometric water meniscus, capillary-condensed between the QTF-AFM tip and the MEMS’ upper plate (inset). (**b)**, Scanning electron micrograph (SEM) image of the fused quartz tip glued to the bottom of the QTF for tapping-mode operation of AFM (inset). (**c**), SEM image of the MEMS force sensor that consists of a parallel-plate capacitor with a variable separation reflecting the force exerted by the capillary water nano-meniscus. (**d**), Determination of the tip-surface contact point *z*_c_ using the effective elasticity *k*_int_ and damping coefficient *b*_int_ of the nano-meniscus obtained by the dynamic atomic force spectroscopy. *z*_c_ represents the position where *k*_int_ shows an abrupt increase from negative to positive values, indicating that the AFM tip does not measure the attractive tension of the meniscus any more, but detect the repulsive hard contact with the MEMS’ upper surface.

Figure 2a presents the experimental scheme of the system consisting of both the quartz-tuning-fork(QTF)-based noncontact AFM (Fig. 2b) that measures accurately the force gradient and the MEMS sensor (Fig. 2c) that measures directly the full-range force^11^. We first produce the nanometric water meniscus that is capillary-condensed as the AFM tip approaches the MEMS surface. The contact point (*z*_c_) is determined as the position where the effective force gradients, elasticity (*k*_int_) and damping coefficient (*b*_int_), show drastic increase (Fig. 2d), where the tip is considered to make a hard contact with the MEMS.

**Figure 3:**
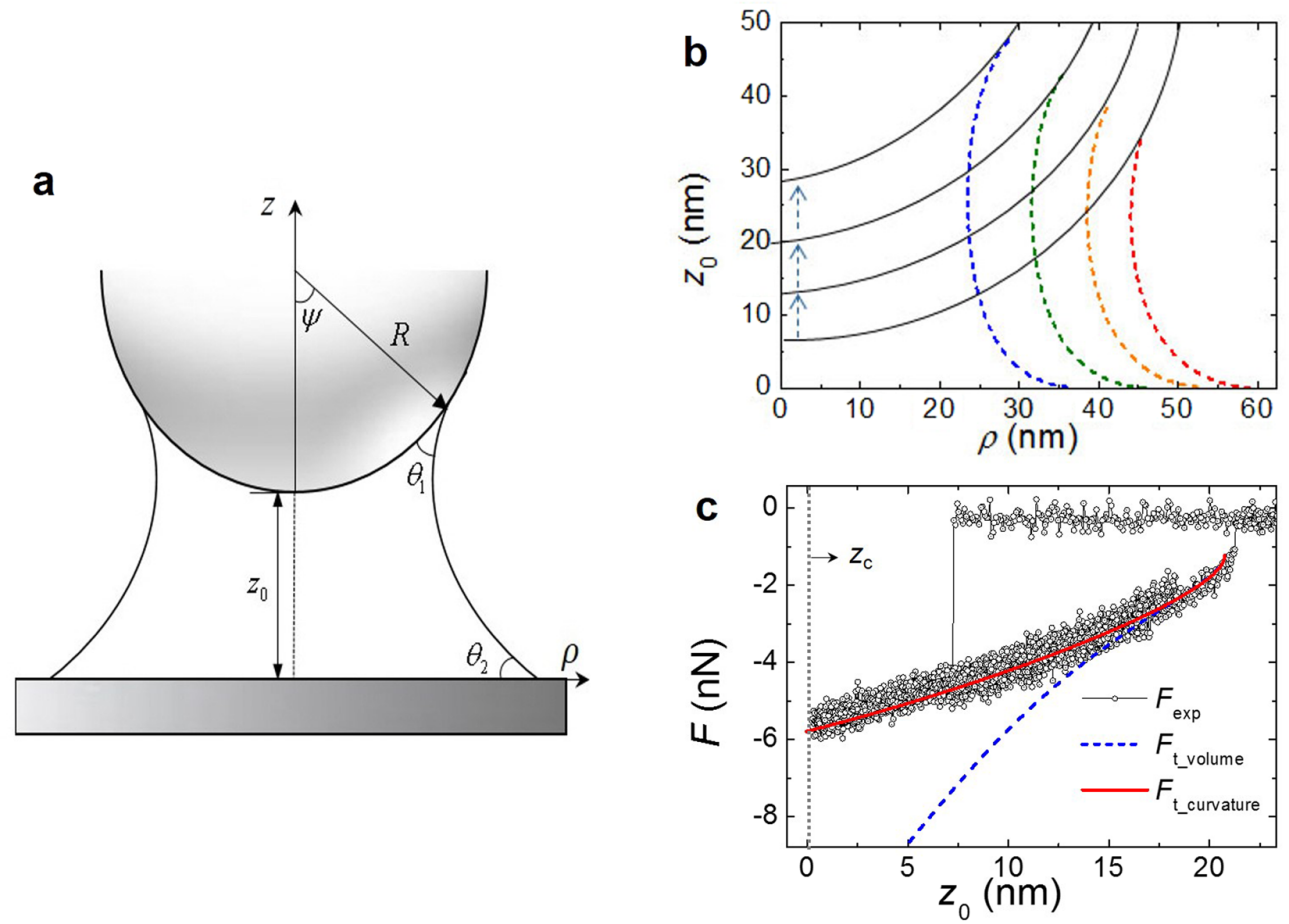
Constant radius-of-curvature of the meniscus at slow retraction. (**a**), Simple representation of the axially symmetric liquid meniscus formed between a spherical tip and a plane surface separated by a distance *z*_0_ with the filling angle *ψ*, the tip’s radius-of-curvature (ROC) *R*, the contact angles *θ*_1_ and *θ*_2_ of water on the two solid surfaces. Here, $\rho$ denotes the cylindrical coordinate. (**b**), Plot of the ROC of the water nano-meniscus *r*_m_, numerically calculated by the Young-Laplace equation, which shows the constant ROC *r*_m_ (or equivalently, constant pressure) during meniscus elongation (from red to yellow to green to blue dotted lines) sufficiently slow enough to allow thermal equilibrium with the gaseous environment. The retraction speed is less than 1 nm/s, which is more than two orders-of-magnitude slower than that of the typical nano-meniscus retraction experiments at constant volume. (**c**), Comparison between experimental results and theoretical calculations for the typical full-range force-distance curve under constant volume (dotted blue) and constant pressure (solid red) conditions. The theoretical results under constant pressure (or constant *r*_m_) show excellent agreement with the experimental data, which justifies the assumption of constant ROC for slow meniscus elongation. Notice that the data presented include the entire capillary force measurements until the tip makes a contact with the surface.

Figure 3a shows the schematic geometry of the capillary meniscus formed in the tip-surface nano-gap. The total capillary force *F*_t_ exerted by the meniscus is calculated by a sum of both the surface-tension force *F*_s_ associated with the three-phase contact line and the capillary-pressure force *F*_p_ caused by the pressure difference between liquid and vapour (see Supplementary S1),

1 $${F}_{{\rm{t}}}={F}_{{\rm{s}}}+{F}_{{\rm{p}}}=2\pi {R}_{{\rm{tip}}}{\gamma }_{{\rm{m}}}[-\sin \,\psi \,\sin (\theta {}_{1}+\psi )+\frac{1}{2{r}_{{\rm{m}}}}{R}_{{\rm{tip}}}\,{\sin }^{2}\psi ]$$

where *R*_tip_ is the tip’s ROC and *γ*_m_ is the effective ST of meniscus. The contact angle *θ*_1_ will be assumed zero along with *θ*_2_ $\approx$ 0 in the case of the hydrophilic silica surfaces (Supplementary S2). To investigate the interaction forces Eq. (1), one can consider constant either the pressure within the meniscus or the volume of the meniscus. The shape of the meniscus can be computed assuming constant volume, which is valid for fast retraction when there is not enough time to reach thermodynamic equilibrium. In this case, the ROC of the meniscus increases to keep the volume constant during tip retraction, while the local surface tension also changes accordingly, as the meniscus is elongated^16^. On the other hand, for slow enough retraction, the ROC remains unchanged due to the stabilized (i.e. thermal equilibrium) pressure difference across the water/vapour interface at constant pressure, and also the surface tension is maintained constant during retraction, both of which can be obtained by best fit of the experimental curves. In our experiment, the retraction speed of the MEMS plate is less than 1 nm/s, more than 10^3^ times slower than that employed to keep the meniscus volume constant^16^. Therefore, in our analysis, we treat the meniscus’ ROC, *r*_m_, as a variable to determine the best fitting value of ST, *γ*_m_, by direct comparison of force measurements and numerical simulations. Note that we assume the capillary force is much higher than the van der Waals force, gravity force (Supplementary S3), and electrostatic force. We ignored the electrostatic force induced by surface charge density in the thin surface hydration-layers since it is about 100 times smaller than the measured force^13^. In particular, we extract the surface tension from Eq. (1) for a water column of at least 7 nm long, whose center is far enough to neglect the influence of electrostatic force. Therefore, we consider that the surface tension we obtain corresponds to the value of the gas-liquid interface at the meniscus equator, rather than the value near the surfaces where surface charge effects may contribute (see also Supplementary S3).

Figure 3b presents four typical (snapshot) configurations of water menisci being elongated during very slow (<1 nm/s) retraction of the MEMS plate for force measurement, where each ROC is numerically evaluated by the Young-Laplace equation^12^. As shown, the initial value of *r*_m_ is maintained constant during retraction until its rupture, that is, the ROC is invariant from red to yellow to green to blue as the tip is retracted. Figure 3c demonstrates the validity of the constant ROC (or constant *r*_m_) condition by quantitative comparison of the measured forces with the theoretical ones that assume either constant curvature (*F*_t_curvature_) or constant volume (*F*_t_volume_). As shown, the experimental force-distance curve matches excellently with the numerical simulation for constant curvature, but deviates severely from that under constant volume^16,17^. Therefore, the isobaric assumption is appropriate for very slow retraction, which also justifies the constant *r*_m_ during the entire force measurement as the meniscus is stretched (Fig. 3b). Notice that in our previously reported time-resolved measurement of activation time for nucleation^18^ as well as rupture^19^ of a water nano-meniscus, each data point was taken at every 10 ms to 20 ms at a fixed position above the substrate, which was long enough to exhibit steady-state response at thermal equilibrium without any abrupt temporal variation of data except at nucleation or rupture. In other words, the speed of the tip (sampling time) was as slow (long) as that of the present work, which justifies our assumption of constant ROC or steady-state state of the nano-meniscus.

**Figure 4:**
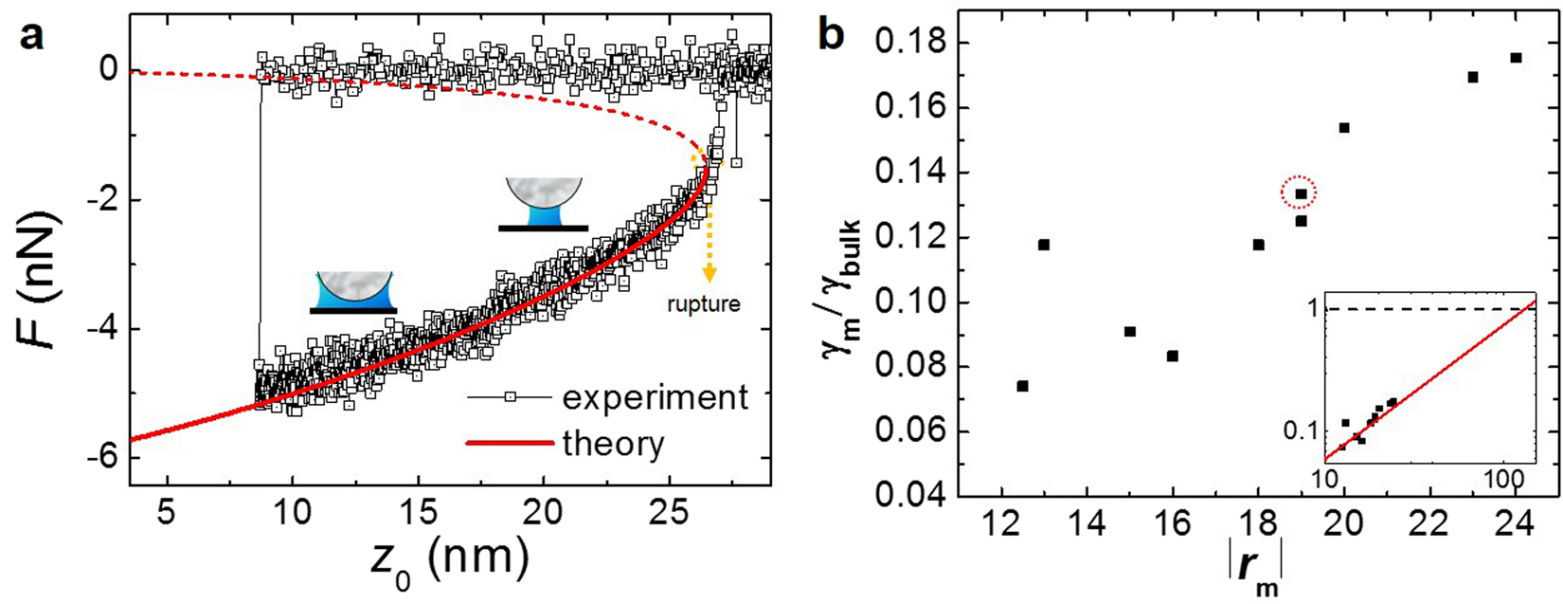
Determination of the best fits for the effective ST, γm, at nanoscale. (**a**), The typical experimental force curve is compared with the numerical solution of Eq. (1) based on the Young-Laplace equation under constant ROC for *R*_tip_ = 50 nm and *θ*_1_ = 0 (supplementary S2), which allows unambiguous determination of the effective ST value γ_m_ as well as the ROC *r*_m_ of the water meniscus. Notice that we have repeated similar force measurement for several menisci of different sizes by varying relative humidity within the condition of constant *r*_m_ without making any direct contact of the tip with the surface, unlike the case of Fig. 3c where the tip contacts the surface before retraction to measure the contact point *z*_c_. (**b**), Ratio of the effective ST, γ_m_/γ_bulk_ (γ_bulk_ is the bulk value at room temperature, 71.97 mN/m) for various values of *r*_m_ (i.e., meniscus sizes) at nanoscale. Each data point represents independent experiments performed for different sizes of the nano-meniscus (the red dotted circle corresponds to the result obtained by the data of Fig. 4a). Notice that the simple extrapolation of the data shown in the inset of 4b expects the bulk-value behaviour of the effective ST at above *r*_m_130 nm.

Figure 4a presents the typical force-measurement result of the MEMS sensor, from which one can determine uniquely *γ*_m_ for a given *r*_m_ such that the best fitting is obtained only for a specific pair of both values (see Supplementary S1). Note that *r*_m_ (meniscus’ radius of curvature) is constant during meniscus experiment and gives the information of *z*_0_ for the rupture distance, from which we can derive the volume of the meniscus according to the Young-Laplace equation for the capillary bridge^12^ having constant *r*_m_ (Supplementary S4). Interestingly, we find the fitted value of *γ*_m_ for *r*_m_ = 19 nm in ambient condition is about 9.59 mN/m. Figure 4b plots various *γ*_m_ for several different *r*_m_ of the menisci, obtained by the same procedures described in Fig. 4a. We have varied relative humidity (~60%) while other conditions are fixed as 1 atm and room temperature (~23.5 °C), each data point in Fig. 4b is given by individual experiment. As observed, *γ*_m_ increases in approximately linear proportion with *r*_m_ up to 25 nm (red circle indicates the data of Fig. 4a) and a simple extrapolation shows the bulk value at ~130 nm ROC (see inset). Figure 4a represents single approach/retraction experiment data for single water meniscus. And Fig. 4b plots each data point of single approach/retraction experiment varying with relative humidity, which determines the size (volume) of the meniscus. Even at single approach/retraction experiment, the meniscus volume can change with respect to variation of z_0_ in equilibrium meniscus in the constant *r*_m_ condition (Supplementary S4).

Let us first justify calculation of the force under the assumption of constant curvature for the meniscus. Equation (1) is calculated by numerical solution of the Young-Laplace equation^11^, which provides the steady-state configuration of the axially symmetric liquid-vapour interface. For fast retraction, the meniscus is not in thermal equilibrium and thus its shape can be computed under constant volume^16^, for which the neck diameter decreases with elongation. However, sufficiently slow retraction leads to the stabilized (equilibrium) constant curvature during stretch (retraction), which is already confirmed by our previous work on the time-resolved experiment while sustaining the signal responses at every moment during retraction^17–19^. Therefore, almost uniform mean *r*_m_ for a nanometrically small tip is calculated in Fig. 3b under the constant curvature condition, as confirmed in Fig. 3c. In our experiment, the retraction speed is below 1 nm/s, more than 1,000 times slower than for the constant-volume experiments^17–19^.

Let us now discuss the treatment of *r*_m_ as a variable. Recent AFM experiments on the capillary water-bridge have shown that *r*_m_ (≈ *z*_0_/2) is much longer than the predicted Kelvin radius at a given relative humidity^11,20^, pronounced especially for water^21–24^, while that obtained by the surface force apparatus agrees well with the Kelvin equation^14,25,26^. This suggests that what we measured is the total forces of the meniscus resulting from capillary condensation as well as other effects such as coalescence due to the pre-adsorbed water layers or the double-layer forces^14^, which provides the information of the ‘effective’ surface tension, rather than the genuine value of surface tension (therefore, in order to investigate any physical change of surface tension of water, one has to separate out the additional effects, which is beyond the scope of our current work and may be considered as a future work). To incorporate this observation in our analysis, we vary *r*_m_ to find the capillary force. Wide variation of *r*_m_, which is numerically calculated for a given rupture distance (or meniscus size), allows one to determine the unique pair of *r*_m_ and *γ*_m_ that best fit the experimental data. For example, *γ*_m_/*γ*_bulk_ ≈ 1/8 when *r*_m_ ≈ 21.6 nm (Fig. 4a). This indicates that since, at the molecular level, ST results from the interface interaction, associated with hydrogen bonding and dispersion force^27,28^, ST exhibits the curvature dependence at nanoscale, as confirmed qualitatively by molecular dynamics simulations^10^. Notice that the expected bulk value at *r*_m_ ~130 nm (inset, Fig. 4b) is consistent with thermodynamics of the curved interface^29^ that predicts the bulk ST above ~100 nm.

Finally, we demonstrate that the curvature-dependent ST resolves the unsettled discrepancies of thermodynamic quantities in various nano-water systems. For example, the calculated tensile strength for vapour-bubble formation by the nucleation-rate theory is about 1,800 atm^3^, much larger than the measured 277 atm^9^. This disagreement can be resolved if the ST assumes 30% of the bulk value, in qualitative agreement with our results as well as with other expected value of 22% in the cluster-formation model of bubble^8,15^ (Supplementary S5). For nano-droplet formation in the supersaturated vapour, the theoretical nucleation rate is underestimated by two orders-of-magnitude compared to experiment, which can be similarly settled by the 30% of bulk ST that produces 4.6-times decrease of the vapour-nucleation energy-barrier, resulting in the 100-fold rate increase^30^. For the water nano-meniscus under the shear-mode AFM that is insensitive to the capillary force, the estimated energy barriers for both capillary condensation and rupture are about 4~5 times smaller^18,19^ than the theoretical values^31^, which can be also resolved by the 20~25% of bulk ST (Fig. 4b), similarly to the nano-droplet formation.

We have quantified the effective ST behaviour at nanoscale by measurement of the total capillary force resulting from the water nano-meniscus, in combination with theoretical calculation of the meniscus ROC based on the Young-Laplace equation. We have shown that the substantially reduced values of the effective ST at nanoscale may resolve the existing discrepancies between experiments and theories associated with the thermodynamic phase-transition effects in three types of nano-confined water. These observations may trigger theoretical development for a better understanding of surface tension at the molecular level. They will allow a novel guidance to the general ST-related phenomena including growth dynamics of liquid nano-clusters^32^ and biological processes in nano-confined liquid^28^, which may further contribute to practical applications such as the ST-controlled self-assembly of bio-molecules and the efficient design of nano-biomaterials^33^ using the surfactant-like effect of ROC.

## Methods

### AFM-MEMS combined system for accurate force measurement

In our system, the QTF-AFM detects the *dynamic* force gradient that provides information on the viscoelasticity of the meniscus^11^, while the MEMS sensor obtains simultaneously the absolute *static* force in the full distance range. The hydrophilic quartz tip, which has an ROC of ~50 nm, is fabricated by a commercial puller (P-2000, Sutter Instruments Co.) and attached to the bottom of QTF for small amplitude-modulation (AM) operation of AFM in the tapping mode^23^.The QTF probe allows stable formation of the water nano-meniscus for high-resolution experiments at a precisely controlled height of the tip, with a quality factor of ~5000, stiffness of 10^3^~10^4^ N/m, resonant frequency of ~32 kHz and driving amplitude of ~1 nm. The AM-AFM measures *k*_int_ given by^20^, ${k}_{int}=\frac{F}{A({z}_{0})}\,\sin \,\theta ({z}_{0})-k(1-\frac{{\omega }^{2}}{{{\omega }_{0}}^{2}})$, where *F* is the driving-force amplitude, *k* the stiffness of the tip, *ω* the drive frequency, *ω*_0_ the resonance frequency, *z*_0_ the tip-sample separation, *A* the tip’s oscillation amplitude and *θ* phase shift of the oscillation. The force measured by AFM is thus ${F}_{{\rm{AFM}}}={\int }_{{z}_{{\rm{r}}}}^{{z}_{0}}dz[\,-\,{k}_{int}]+{F}_{0}$, where *z*_r_ is the rupture distance and *F*_0_ is a constant, whereas the total capillary force that the MEMS detects is simply ${F}_{MEMS}=-\,{k}_{MEMS}{\rm{\Delta }}x$, where Δ*x* is the meniscus-induced displacement of the MEMS’ upper plate (made of silica) and the stiffness is *k*_MEMS_ = 2.43 ± 0.01 N/m that is about 10 times stiffer than the water meniscus^11^. Notice that the two values, *F*_MEMS_ and *F*_AFM_, have been shown to provide exactly the same results^11^ (details described in the reference) by a proper assignment of the integration constant *F*_0_.

## Electronic supplementary material

Supplementary Information

## References

[1] Moody MP, Attard P (2003). Curvature-dependent surface tension of a growing droplet. Phys. Rev. Lett..

[2] Laaksonen A, Talanguer V, Oxtoby DW (1995). Nucleation: Measurements, theory, and atmospheric applications. Annu. Rev. Chem..

[3] Fisher JC (1948). The Fracture of Liquids. J. Appl. Phys..

[4] Tolman RC (1948). Consideration of the Gibbs Theory of Surface Tension. J. Chem. Phys..

[5] Koenig JO (1950). On the Thermodynamic Relation between Surface Tension and Curvature. J. Chem. Phys..

[6] Hirschfelder, J. O., Curtis, C. F. & Bird, R. B. Molecular Theory of Gases and Liquid. Wiley, New York (1954).

[7] Zhang R, Khalizov A, Wang L, Hu M, Xu W (2012). Nucleation and Growth of Nanoparticles in the Atmosphere. Chem. Rev..

[8] Fowkes FM (1964). Attractive forces at interfaces. Ind. Eng. Chem. Res..

[9] Briggs LJ (1950). Limiting Negative Pressure of Water. J. Appl. Phys..

[10] Liu H, Cao G (2016). Effectiveness of the Young-Laplace equation at nanoscale. Sci. Rep..

[11] Kwon S, Stambaugh C, Kim B, An S, Jhe W (2014). Dynamic and static measurement of interfacial capillary forces by a hybrid nanomechanical system. Nanoscale.

[12] Orr FM, Scriven LE, Rivas AP (1975). Pendular rings between solids: meniscus properties and capillary force. J. Fluid Mech..

[13] Israelachvili, J. Intermolecular and Surface Forces. Academic Press, New York (2011).

[14] Fisher LR, Israelachvili JN (1980). Determination of the Capillary pressure in menisci of molecular dimensions. Chem. Phys. Lett..

[15] Kwak HY, Oh SD (2004). Gas–vapor bubble nucleation—a unified approach. J. Colloid. Interf. Sci..

[16] Willett CD, Adams MJ, Johnson SA (2000). & Seville, J. P. K. Capillary Bridges between Two Spherical Bodies. Langmuir.

[17] Sirghi L, Szoszkiewicz R, Riedo E (2006). Volume of a nanoscale water bridge. Langmuir.

[18] Sung B, Kim J, Stambaugh C, Chang S, Jhe W (2013). Direct measurement of activation time and nucleation rate in capillary-condensed water nanomeniscus. Appl. Phys. Letts..

[19] Bak W, Sung B, Kim J, Kwon S, Kim B, Jhe W (2015). Time-resolved observation of thermally activated rupture of a capillary-condensed water nanobridge. Appl. Phys. Letts..

[20] Lee M, Sung B, Hashemi N, Jhe W (2009). Study of a nanoscale water cluster by atomic force microscopy. Faraday Discuss..

[21] van Honschoten JW, Brunets N, Tas NR (2010). Capillarity at the nanoscale. Chem. Soc. Rev..

[22] Weeks BL, Vaughn MW, DeYoreo JJ (2005). Direct imaging of meniscus formation in atomic force microscopy using environmental scanning electron microscopy. Langmuir.

[23] Kim B, Kwon S, Mun H, An S, Jhe W (2014). Energy dissipation of nanoconfined hydration layer: Long-range hydration on the hydrophilic solid surface. Sci. Rep..

[24] Bartošík M (2017). Nanometer-Sized Water Bridge and Pull-Off Force in AFM at Different Relative Humidities: Reproducibility Measurement and Model Based on Surface Tension Change. J. Phys. Chem. B.

[25] Kohonen MM, Christenson HK (2000). Capillary condensation of water between rinsed mica surfaces. Langmuir.

[26] Fisher LR, Gamble RA, Middlehurst J (1981). The Kelvin equation and the capillary condensation of water. Nature.

[27] Galamba N, Cabral B (2008). J., C. The changing hydrogen-bond network of water from the bulk to the surface of a cluster: a Born-Oppenheimer molecular dynamic study. J. Am. Chem. Soc..

[28] Cioci F (1996). Effect of Surface Tension on the Stability of Heat-Stressed Proteins: A Molecular Thermodynamic Interpretation. J. Phys. Chem..

[29] Guggenheim EA (1940). The thermodynamics of interfaces in systems of several components. Trans. Faraday Soc..

[30] Strey R, Wagner PE, Viisanen Y (1994). The problem of measuring homogeneous nucleation rates and the molecular contents of nuclei: Progress in the form of nucleation pulse measurement. J. Phys. Chem..

[31] Men Y, Zhang X, Wanga W (2009). Capillary liquid bridges in atomic force microscopy: formation, rupture, and hysteresis. J. Chem. Phys..

[32] Yunker PJ, Still T, Lohr MA, Yodh AG (2011). Suppression of the coffee-ring effect by shape-dependent capillary interactions. Nature.

[33] Barbosa O, Ortiz C, Berenguer-Murcia A, Torres R, Rodrigues RC, Fernandez-Lafuente R (2014). Glutaraldehyde in bio-catalysts design: a useful crosslinker and a versatile tool in enzyme immobilization. RSC Adv..

