# Supplementary material for: Adhesive force measurement of steady-state water nano-meniscus: Effective surface tension at nanoscale
Source: Sci Rep. 2018 May 31;8:8462. doi: 10.1038/s41598-018-26893-5 (PMC5981305; doi:10.1038/s41598-018-26893-5)
Supplement: Supplementary file 1 — Supplementary Information [file 41598_2018_26893_MOESM1_ESM.pdf]

Supplementary information

"Adhesive force measurement of steady-state water nano-meniscus: Effective surface tension at nanoscale"

Soyoung Kwon[1], Bongsu Kim[1], Sangmin An[1], Wanhee Lee[1], Ho-Young Kwak[2], and Wonho Jhe[1]*

S1. Numerical simulation of Eq. (1) and its validity of fitting procedures

The complete system of equations utilized in fitting the experimental data on numerical simulation of Eq. (1) and its validity of the fitting procedures are described. Figure S1 shows the solution of Young-Laplace equation with general expressions for the principal curvatures of any surface of revolution with boundary conditions. And Fig. S2 shows the strategy of numerical solution of the capillary force. The configuration of a liquid/vapour interface at rest is described by the Young-Laplace equation that relates the difference in hydrostatic pressure across the interface to ROC r_m and surface tension γ_m. The nanometric water meniscus formed in between the AFM probe and the MEMS surface is axisymmetric and r_m satisfies the first equation of Fig. S1. Since r and z are the cylindrical coordinates of the meniscus and ε is the angle between the normal to the meniscus and the vertical axis, it reduces to the second equation of Fig. S1. This equation should be solved as the two-point boundary-value problem for which the boundary conditions are the inclinations determined by the slopes of the solid surfaces and the contact angles θ_1 and θ_2. Thus the boundary conditions are given as the third equations of Fig. S1 with the filling angle ψ. The boundary-value problem then give the solutions (4th and 5th equations of Fig. S1) with the curvature-dependent parameter c. The force is computed for all the filling angles ψ during the entire retraction ($z_0 > 0$), where the distance z_0 can be evaluated by numerical solution of the

Young-Laplace equation for a given ψ. Figure S2 provides the detailed successive fitting procedures.

Solution of Young-Laplace equation

General expressions for the principal curvatures of any surface of revolution

$$\frac{1}{r_m} = \frac{d^2z/dr^2}{[1+(dz/dr)^2]^{\frac{3}{2}}} + \frac{dz/dr}{r[1+(dz/dr)^2]^{\frac{1}{2}}}$$

With dimensionless variables

$$y = z/R, \quad x = r/R$$

$$u = -\sin\epsilon, \quad dy/dx = \tan\epsilon$$

$$R/r_m = -du/dx - u/x$$

Boundary conditions

$$\left\{ \begin{array}{lll} u_2 = -\sin(\pi-\theta_2), & y_2 = 0, & \\ u_1 = -\sin(\theta_1+\psi), & y_1 = z_0 + 1 - \cos\psi, & x_1 = \sin\psi \end{array} \right\}$$

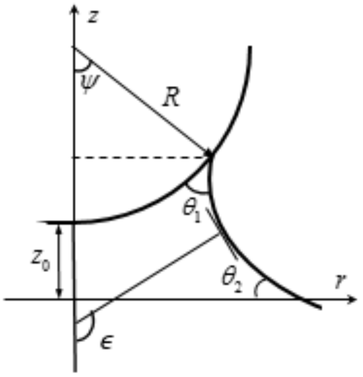

F. M. Orr *et al. J. Fluid Mech.* 67, 723 (1975).
J. C. Melrose, *A.I.Ch.E. J.* 12, 986 (1966)

The boundary-value problem has the solution

$$x = -r_m[u \mp (u^2+c)^{\frac{1}{2}}]$$

$$c \equiv \left(\frac{R}{r_m}\sin\psi\right)^2 - 2\frac{R}{r_m}\sin\psi\sin(\theta_1+\psi)$$

$$y = \frac{r_m}{R}\int_{u_2}^{u}\left(\frac{u\,du}{(1-u^2)^{\frac{1}{2}}}\,\frac{u \mp (u^2+c)^{\frac{1}{2}}}{\mp(u^2+c)^{\frac{1}{2}}}\right)$$

$$= \frac{r_m}{R}\left\{-\cos\epsilon - \cos\theta_2 - \frac{1}{k}[E(\Phi_2,k) - E(\Phi,k)] + \frac{1-k^2}{k}[F(\Phi_2,k) - F(\Phi,k)]\right\}$$

$$k \equiv 1/(1+c)^{\frac{1}{2}}, \quad \phi_2 = \theta_2 - \frac{1}{2}\pi, \quad \phi = \frac{1}{2}\pi - \epsilon,$$

$E(\Phi,k)$: Elliptic integral of the second kind, $F(\Phi,k)$: Elliptic integral of the first kind

Figure S1 | Solution of the Young-Laplace equation with general expressions for the principal curvatures of any surface of revolution under given boundary conditions.

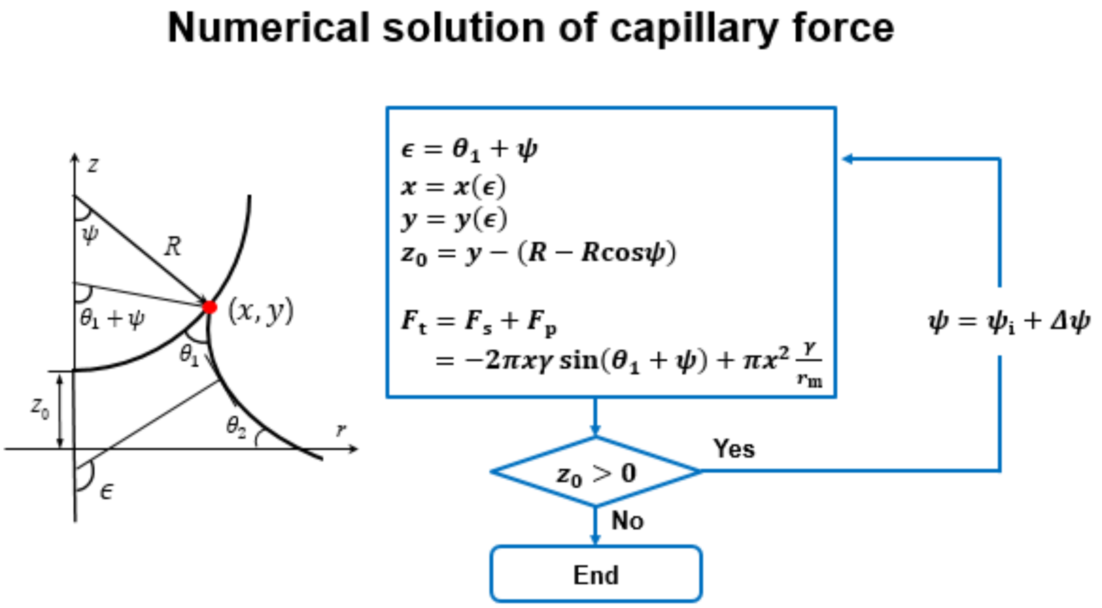

Figure S2 | Numerical solution of capillary force. The force is computed for all the filling angles ψ during the entire retraction (z_0 > 0), where the distance z_0 can be evaluated by numerical solution of the Young-Laplace equation for a given ψ.

Figure S3 presents typical calculation results of the total capillary adhesion force F_t (Eq. 1 in the context) under the assumption of constant curvature, which can be used to determine the unique best-fitting effective ST value of the water nano-meniscus, $\gamma_{m,}$, as well as its corresponding ROC, r_m. The force is computed for all the filling angles ψ during the entire tip retraction (z_0 >0), where the tip-sample distance z_0 can be evaluated by numerical solution of the Young-Laplace equation for a given ψ, so that quantitative comparison of the measured force with the theoretical one can be made as a function of z_0. Here, we discuss the validity of our fitting procedures. Firstly, the contact angles θ_1 and θ_2 are assumed zero for the hydrophilic silica surfaces, which is justified in the following two ways: (i) Even if we use nonzero values of θ_1 (e.g., 10 or 20 degree), we only find slightly roughly fitted curves, which nonetheless still predict the same value of γ_m as that for θ_1=0, while there is a slight (~20%) increase of r_m. (ii) Use of the three-phase contact-line tension of the silicon dioxide surface ($\approx 10^{-9}$ J/m[S1,S2]) leads to the simple estimate that the contact angle becomes zero

when the ROC of meniscus r_m is less than ~120 nm. Secondly, even when we include an error in the value of R in our numerical simulations, we find only a slight change of γ_m; a 10% increase (decrease) of R results in a 10% decrease (increase) of γ_m, accompanied by about 3% increase (decrease) of r_m.

Figures S3a and b show the numerical results of F_t versus z_0 for several trial values of the meniscus ROC, r_0, while the value of γ_m is fixed as the bulk value (72.0 mN/m). As can be seen, the larger r_0 is associated with the larger z_0, where the maximum z_0 indicates the rupture distance that can be determined experimentally. The total force F_t, normalized to each numerical contact force F_0 at $z_0 = 0$, is plotted in Fig. S3b. Figure S3c presents the force F_t versus several different trial values of ST, γ_0, for a given maximum value of z_0 (or r_m), that is, for a given meniscus size characterized by the rupture distance z_r. We observe that the magnitude of F_t increases in a linear proportion to the ST γ_0 (see Eq. (1)) while the force-distance behaviour maintains the same qualitative shape, as indicated by the identical normalized curve in Fig. S3d. As expected, the numerical solution does not exist for the distance z_0 beyond z_r, where z_r corresponds to the measured rupture position of the water nanomeniscus, so that no water meniscus satisfying the Young-Laplace equation exists. In Fig. S3, the dashed part of each curve corresponds to the physically meaningless solution since the solution prior to the rupture point violates the fact that a decrease of the tip-sample distance causes a decrease of the activation energy barrier that leads to a bigger water meniscus[S3]. As a consequence, simultaneous variation of r_0 and γ_0 then allows a unique and unambiguous determination of the meniscus' ST γ_m as well as the corresponding r_m for a given meniscus size (or rupture distance).

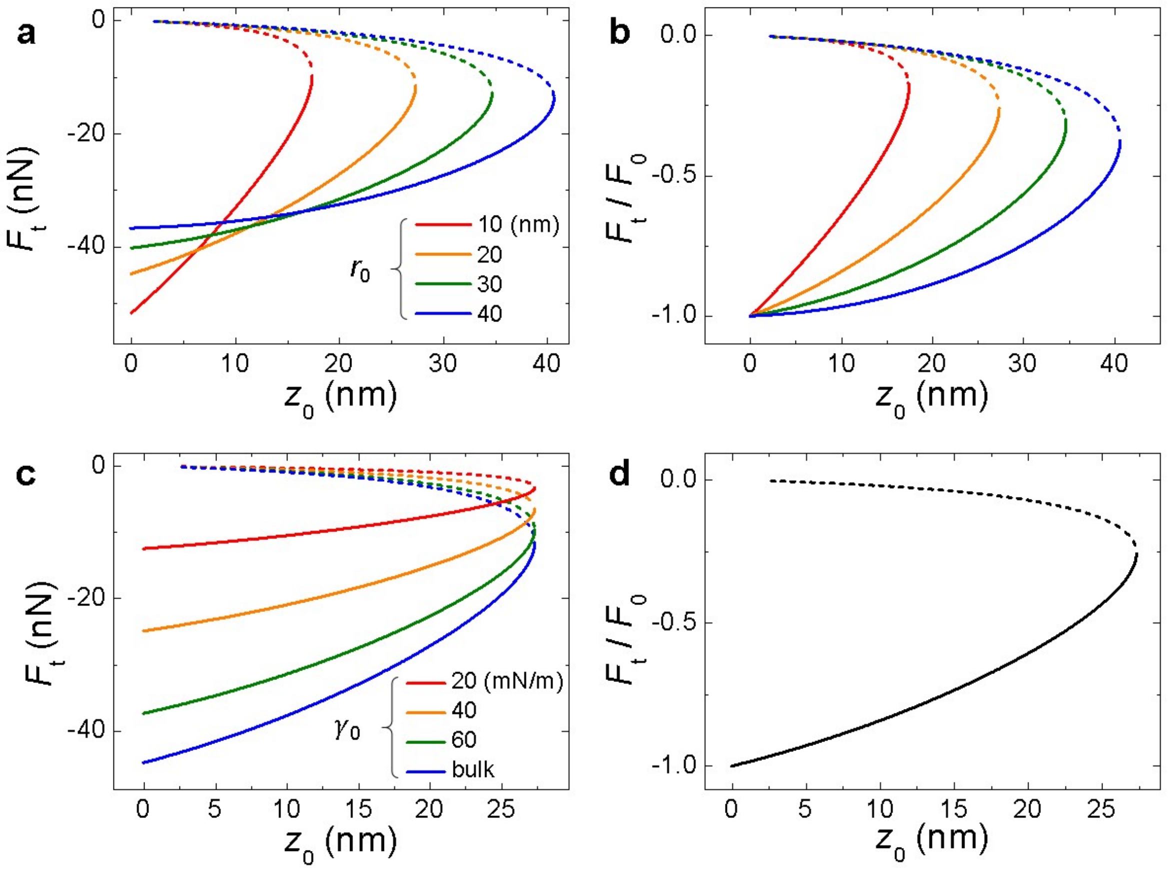

Figure S3 | Simulation results of the total force F_t versus the tip-sample distance z_0. The total capillary force F_t can be calculated if one knows the values of the meniscus' effective ST γ_m and the filling angle ψ (Eq. (1)). For a given ψ, the corresponding value of z_0 can be evaluated by numerical solution of the Young-Laplace equation for each r_m value. **a**, **b** show the theoretical results of the force-distance curve for several trial values of the meniscus ROC, r_0, at a fixed value of γ_m (e.g., bulk value of 72.0 mN/m) and the corresponding normalized force curves, respectively. As shown, the maximum value of z_0 increases linearly with r_0, indicating the larger rupture distance for the larger ROC. **c**, **d** show the plots of the force-distance curve for different trial values of ST, γ_0, at a fixed value of r_m and the normalized results that fit into a single universal curve, respectively. Notice that plots of **b** and **d** are normalized to the contact adhesive force F_0 evaluated at z_0=0. The numerical simulations were performed for the spherical AFM tip's ROC R of 50 nm. Then, the best fit with the experimental results provides the uniquely determined pair values of r_m and γ_m for a given size of the nano-meniscus that is characterized by its rupture distance.

S2. Assumption of zero θ_1 and θ_2 for the hydrophilic silica surfaces

Equation (1) is the total interaction force calculated with θ_1 which is the contact angle between the liquid meniscus and the AFM probe. And the total capillary force F_t is also denoted with the contact angle between the liquid meniscus and the MEMS substrate, θ_2 (Fig. S4).

According to the numerical analysis the force can be calculated for either the tip or the plate and the same result obtained for the total capillary force, and we have done the numerical simulation with Eq. (1) including θ_1. With numerical simulation of Eq. (1) and its validity of fitting procedures (Supplementary S1), the contact angles θ_1 and θ_2 are assumed zero for the hydrophilic silica surfaces. That is justified in the following two ways: (i) Even if we use nonzero values of θ_1 (e.g., 10 or 20 degree), we only find slightly roughly fitted curves, which nonetheless still predict the same value of γ_m as that for $\theta_1 = 0$, while there is a slight (~ 20%) increase of r_m. (ii) Use of the three-phase contact-line tension of the silicon dioxide surface (10^{-9} J/m) leads to the simple estimate that the contact angle becomes zero when the ROC of meniscus rm is less than ~ 120 nm. Notice that, even when we include an error in the value of R in our numerical simulations, we find only a slight change of γ_m; a 10% increase (decrease) of R results in a 10% decrease (increase) of γ_m, accompanied by about 3% increase (decrease) of r_m.

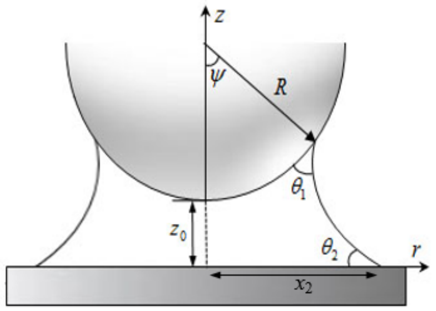

Surface tension force

$$F_s = -2\pi R\gamma \sin\psi \sin(\theta_1 + \psi) \quad \text{or} \quad -2\pi x_2 \gamma \sin\theta_2$$

Capillary pressure force

$$F_p = \frac{\gamma}{r_m}\pi R^2 \sin^2\psi \quad \text{or} \quad \frac{\gamma}{r_m}\pi x_2^2$$

Total interaction force

$$F_t = F_s + F_p = 2\pi R\gamma[-\sin\psi \sin(\theta_1 + \psi) + \frac{1}{2r_m} R \sin^2\psi]$$
$$= 2\pi x_2 \gamma[-\sin\theta_2 + \frac{1}{2r_m} x_2]$$

ψ : Filling angle
R : AFM tip radius of curvature
γ : Surface tension of the interface
θ_1 : Water contact angle of solid surface
r_m : Radius of curvature of the meniscus

Figure S4 | Total interaction force including surface tension force and capillary pressure force.

S3. Assumption for gravity effect and surface charge effect

We measured the capillary force at the ambient condition under 60% relative humidity between hydrophilic surfaces. The surfaces have the effect of gravity, but these forces are negligible since the capillary force is much stronger than these. We assume the water meniscus formed between the tip and the MEMS surface as the simple form of a cylinder (radius R) with maximum volume (i.e., light blue region schematically drawn in Fig. S5), even though the actual water bridge that satisfies the Laplace-Young equation has a smaller volume at the mean curvatures[S4] as shown by the enclosed region coloured in dark blue. The maximum gravity force due to the cylindrical volume is $\sim 2\times 10^{-18}$ N (calculated with the tip radius, for example, $R(\geq r)$ = 58 nm, h = 7 nm, and density of liquid water = 1,000 kgm^{-3}) while the experimentally obtained capillary force is a few nN which is 10^{9} times greater than the effect of water gravity. Therefore, the effect of gravity is negligible in this nanoscale water-bridge experiment.

Concerning possible surface charge effect, it has been known that any surface charge produced during fresh cleavage of a mica sheet disappears within several minutes in ambient condition, while it remains for a day or so in vacuum[S5]. Nonetheless, it will be interesting to investigate the effect of surface charge because it changes the structure of water near the surface by re-orienting its dipoles in a narrow layer adjacent to the probe and plate surfaces, which may contribute to surface tension near the surface depending on the fraction of volume of water layer.

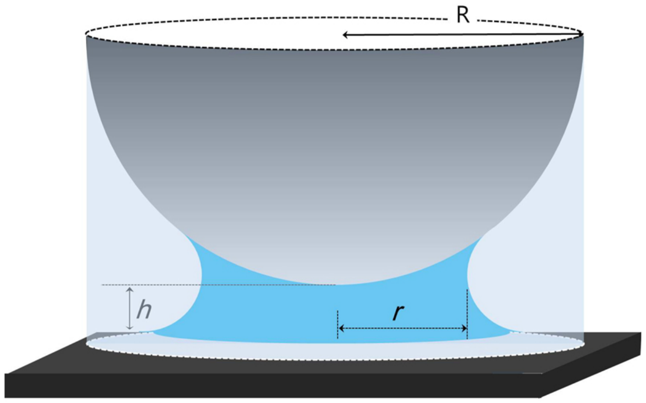

Figure S5 | Maximized volume of water meniscus for calculating gravity force. We assume gravity effect is almost negligible with calculation of gravity force by assumption of maximized volume of water.

S4. Meniscus volume with respect to r_m

We calculated the meniscus volume variation with respect to tip position information (z) indicating the meniscus stretching or retraction in condition of constant r_m. We checked four different constant r_m (10 nm, 15 nm, 20 nm, 25 nm) which varied by changing of relative humidity. As the tip stretches (retracts), the volume of meniscus increases until middle region and decreases until the rupture event is performed. Also the volume of meniscus increases with increment of r_m in both cases of tip radius, 25 nm (Fig. S6a) and 50 nm (Fig. S6a).

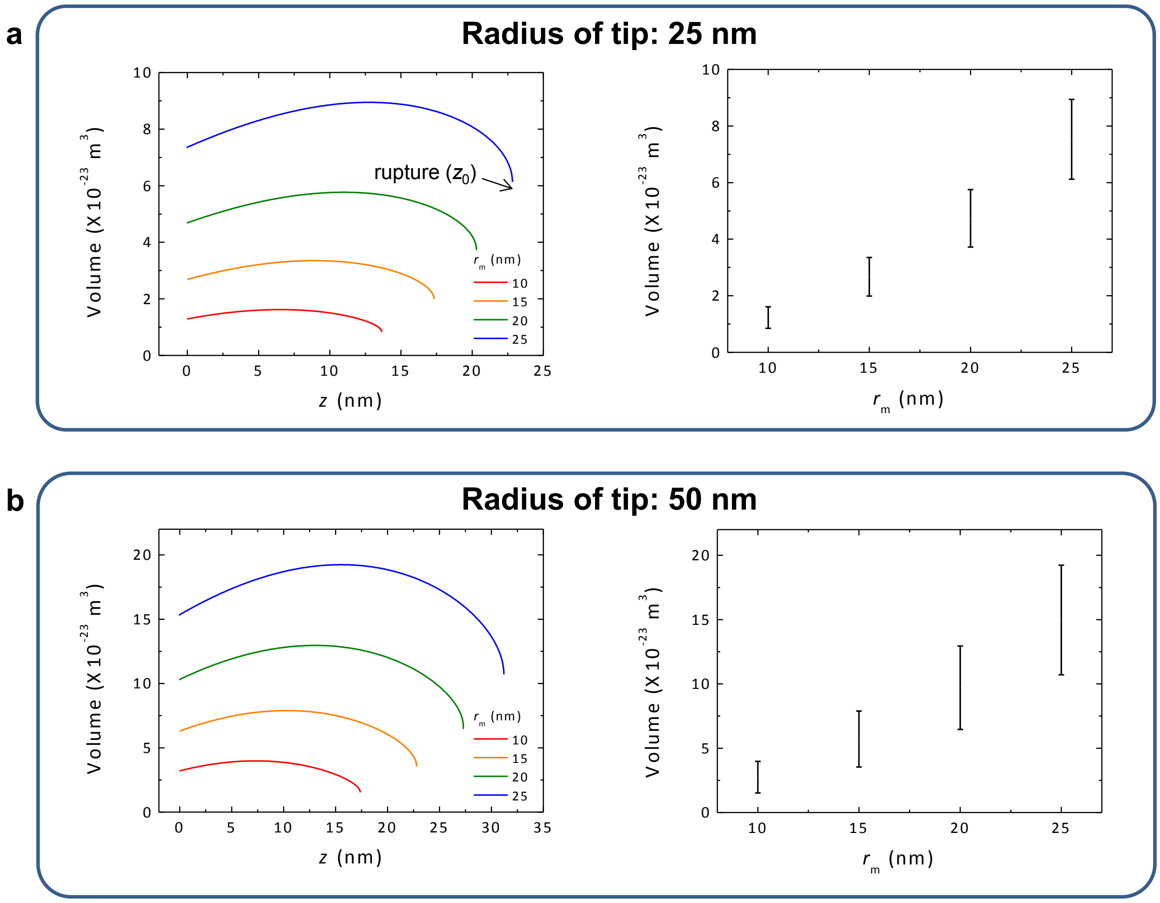

Figure S6 | Calculated meniscus volume with constant r_m. We derived the meniscus volume with respect to z information in condition of four different constant r_m which varied by a relative humidity. As the tip stretches (retracts), the volume of meniscus changes and also rm dependence is depicted in two cases of tip radius, **a,** 25 nm and **b,** 50 nm.

S5. Theories of nanobubble formation and estimates of surface tension

Here we consider two theories in regard to formation of nanobubble and show how the aforementioned discrepancies between theory and experiment can be resolved by the substantially reduced ST at nanoscale. First, the theory of bubble nucleation predicts the nucleation rate (J) of vapour bubbles[S6] given by,

$$J = \frac{Nk_{\mathrm{B}}T}{h}\exp\left[-\left(\Delta f_0^* + \frac{16\pi\gamma^3}{3p^2}\right)\Big/k_{\mathrm{B}}T\right], \tag{S1}$$

where Δf_0^* is the free energy of activation, p the pressure, k_{B} the Boltzmann constant, h the Planck constant and γ the surface tension of the nanobubble. Then the bubble nucleates at a minimum rate by first fracturing the liquid water at the threshold negative pressure p (assuming Δf_0^* is zero for the smallest value of p),

$$p = \left[\frac{16\pi\gamma^3}{3(k_{\mathrm{B}}T\ln Nk_{\mathrm{B}}T/h)}\right]^{1/2}. \tag{S2}$$

The calculated pressure from Eq. (S2) (1,778 atm), which assumes the bulk value of ST, is about 6 times larger than the experimental value (277 atm)[S7]. This disagreement may be reconciled if the ST at nanoscale is in fact smaller than the macroscopic value. For example, from the relation $\gamma \propto p^{2/3}$, the pressure obtained by the nucleation theory agrees qualitatively with the measurement if γ having about 30 % of the bulk value is considered.

Second, let us consider the cluster model for bubble formation that provides the expression of the surface tension as follows[S8],

$$\gamma = \frac{\mathrm{r}}{2}\left(\frac{12\varepsilon}{3v_0}\right). \tag{S3}$$

Here r is the radius of a liquid water molecule, v_0 the effective molecular volume of the liquid, and ϵ the energy to separate a pair of molecules from an intermolecular distance of d_0 to the critical distance d_c (i.e. the mean distance between molecules at the critical point)[S9],

$$\varepsilon = -4\varepsilon_0\left[1-\frac{\rho_c}{\rho_0}\right]\left[\left(\frac{d}{d_0}\right)^6-\left(\frac{d}{d_0}\right)^{12}\right], \qquad \text{(S4)}$$

where, ρ_c is the critical density, ρ_0 the liquid density, E_I the ionization potential and α the polarizability of a liquid molecule. From Eqs. (S3) and (S4), one can calculate the ST value γ to be about 22% of the bulk, which is consistent with the results presented in our manuscript.

References

S1. J. K. Berg, C. M. Weber, H. Riegler, Impact of Negative Line Tension on the Shape of Nanometer-Size Sessile Droplets. *Phys. Rev. Lett.* 105, 076103 (2010).

S2. D. Aronov, G. Rosenman, Z. Barkay, Wettability study of modified silicon dioxide surface using environmental scanning electron microscopy. *J. Appl. Phys.* 101, 084901 (2007).

S3. E. Sahagun, P. Garcia-Mochales, G. M. Sacha, and J. J. Saenz, Energy Dissipation due to Capillary Interactions: Hydrophobicity Maps in Force Microscopy. *Phys. Rev. Lett.* 98, 176106 (2007).

S4. F. M. Orr, L. E. Scriven, A. P. Rivas, Pendular rings between solids: meniscus properties and capillary force. *J. Fluid Mech.* 67, 723 (1975).

S5. Lei Xu, Anna Lio, Jun Hu, D. Frank Ogletree, and Miquel Salmeron, Wetting and Capillary Phenomena of Water on Mica, *J. Phys. Chem. B* 102, 540-548 (1998).

S6. J. C. Fisher, The Fracture of Liquids. *J. Appl. Phys.* **19**, 1062 (1948).

S7. L. J. Briggs, Limiting negative pressure of water. *J. Appl. Phys.* **21**, 721 (1950).

S8. H. Y. Kwak, S. D. Oh, Gas-vapor bubble nucleation- a unified approach, *J. Colloid and Interface Sci.* 278, 436 (2004).

S9. H. Y. Kwak, R. L. Panton, Tensile strength of simple liquids predicted by a model of molecular interactions. *J. Phys. D: Appl. Phys.* 18, 647 (1985).
